# BITS 2015: the annual meeting of the Italian Society of Bioinformatics

**DOI:** 10.1186/s12859-016-1187-2

**Published:** 2016-11-08

**Authors:** Luciano Milanesi, Alessandro Guffanti, Giancarlo Mauri, Marco Masseroli

**Affiliations:** 1 CNR, Institute of Biomedical Technology, Milan, Italy; 2 Genomnia srl, Milan, Italy; 3 University of Milano-Bicocca, Milan, Italy; 4 Politecnico di Milano, Milan, Italy

**Keywords:** BITS, Bioinformatics, Italian Society of Bioinformatics meeting

## Abstract

This preface introduces the content of the BioMed Central journal Supplements related to the BITS 2015 meeting, held in Milan, Italy, from the 3^th^ to the 5^th^ of June, 2015.

## BITS, the Italian Society of Bioinformatics

BITS, the Italian Society of Bioinformatics [1], is the largest non-profit association of researchers involved in Bioinformatics with work activities or interest in Italy. The primary aim of BITS is to join the research scientists interested in Bioinformatics, meant as a multi-disciplinary science for the study of biological systems at the molecular and cellular level by using informatics and computational methods and models. Main goals of the association are the study, development and spreading of Bioinformatics in the scientific, academic, technological and industrial environment. Since its foundation, BITS has continuously increased the number of members and was recognized as a Regional group of the International Society for Computational Biology (ISCB). BITS promotes activities as courses and workshops, at national and international level. Such events were mainly located in Italy and, in some cases, abroad.

## BITS 2015 annual meeting

The twelfth BITS meeting has been held in Milan, from the 3^th^ to the 5^th^ of June, 2015. The meeting was organized by Luciano Milanesi, Giancarlo Mauri and Marco Masseroli, together with a Scientific Committee including most of the Italian Bioinformatics senior scientists. BITS 2015 has been organized during the EXPO 2015, and included the thematic workshop “WHEAT FOR THE FUTURE: Advancing wheat research for global food security” held at the EXPO 2015 Italian Pavilion on June 3^rd^ at 14:00. The EXPO 2015 offered the possibility to the BITS 2015 participants to visit the exhibitions of the 140 countries offering a concrete answer to a vital need: being able to guarantee healthy, safe and sufficient food for everyone, while respecting the Planet and its equilibrium. About 250 participants attended the BITS 2015 meeting, the highest number over the past BITS meetings. The scientific program included the following keynote speakers:

- Prof. Charles CANTOR (Boston University, Boston, USA)
- Prof. Daniele CUSI (University of Milan, Milan, Italy)
- Prof. Marialuisa LAVITRANO (University of Milano-Bicocca, Milan, Italy)
- Dr. Niklas BLOMBERG (ELIXIR Director, United Kingdom)
- Dr. Jan G. BJAALIE (INCF Neuroinformatics Board & University of Oslo, Norway)

Participants were invited to submit scientific contributions either as oral presentations or posters. After evaluation of the 132 abstracts received, the Scientific Committee (Table 1) selected 23 of them for oral presentations, and accepted 109 as posters.

**Table 1:** BITS 2015 Program committee

Claudia Angelini	CNR, IAC, Napoli, Italy
Roberta Bosotti	Nerviano Medical Sciences, Nerviano (MI), Italy
Raffaele Calogero	University of Torino, Italy
Rita Casadio	University of Bologna, Italy
Michele Caselle	University of Turin, Turin, Italy
Alessandro Cestaro	Fondazione E. Mach, San Michele all’Adige, Trento, Italy
Federica Chiappori	CNR, Institute for Biomedical Technologies, Milan, Italy
Angelo Facchiano	CNR, Institute of Food Science, Avellino, Italy
Giorgio Grillo	CNR, Institute for Biomedical Technologies, Bari, Italy
Alessandro Guffanti	Genomnia srl, Milano, Italy
Manuela Helmer-Citterich	University of Rome “Tor Vergata”, Italy
Giovanni Lavorgna	Ospedale San Raffaele, Milan, Italy
Vito Flavio Licciulli	CNR, Institute for Biomedical Technologies, Bari, Italy
Sabino Liuni	CNR, Institute for Biomedical Technologies, Bari, Italy
Paolo Magni	University of Pavia, Italy
Anna Marabotti	University of Salerno, Italy
Roberto Marangoni	University of Pisa, Italy
Pier Luigi Martelli	University of Bologna, Bologna, Italy
Marco Masseroli	Politecnico di Milano, Italy
Giancarlo Mauri	University of Milano Bicocca, Italy
Luciano Milanesi	CNR, Institute for Biomedical Technologies, Milan, Italy
Heiko Muller	IIT –Istituto Italiano di Tecnologia, Genoa, Italy
Alessandro Pandini	Brunel University London, UK
Giulio Pavesi	University of Milan, Milan, Italy
Mattia Pellizzola	IIT –Istituto Italiano di Tecnologia, Genoa, Italy
Marco Pellegrini	CNR - Istituto di Informatica e Telematica, Pisa, Italy
Graziano Pesole	CNR - Institute of Biomembranes and Bioenergetics, Bari, Italy
Paolo Romano	IRCCS San Martino IST, Genoa, Italy
Roberto Tagliaferri	University of Salerno, Italy
Luigi Varesio	Giannina Gaslini Institute, Genoa, Italy

After the selection of the scientific contributions, the Conference program was organized into the following sessions: Omics; Synthetic and Systems Biology; Algorithms for Bioinformatics; Macromolecular Structure; Stochastic Modelling; Biological Databases; Genomics, Transcriptomics and NGS; Management, Integration and Analysis of Clinical and Biomolecular Data; Metagenomics; Molecular Evolution; Pharmacogenomics; Protein Structure and Function; Proteomics.

## BITS 2015 supplement to BMC Bioinformatics journal

All the Authors of the scientific contributions that were presented at the meeting in form of either oral presentation or poster were invited, after the meeting, to prepare and submit a manuscript to be evaluated for publication in a BioMed Central Bioinformatics journal Supplement. The Editor of the Supplement, Luciano Milanesi, designated by the Bioinformatics Italian Society Steering Committee, invited Marco Masseroli and Alessandro Guffanti, members of the Scientific Committee, to serve as Associate Editors for the handling of the submitted manuscripts. Manuscripts were peer-reviewed by at least two external well-known international scientists, in agreement with BMC rules for supplements’ manuscripts evaluation.

## Evaluation process outline

As for previous BioMed Central journal supplements for BITS meetings, a peer-reviewed careful evaluation of the submitted manuscripts has been performed. Each manuscript was assigned to an Associate Editor, on the basis of specific expertise and, when possible, by considering the previous involvement in the contribution selection process. Any conflict of interest was of course avoided.

The Associate Editor preliminarily evaluated the manuscript and assigned it to at least two international independent reviewers, selected from international literature or personal contact, on the basis of their specific expertise.

In case of Reviewers’ negative comments, the manuscript was rejected. In case of favourable Reviewers’ evaluations, their comments were sent to the Authors with the request of submitting a major/minor revised version of their manuscript within two months.

The revised version of the manuscript was then further evaluated. In case only minor revision was recommended, the Supplement Editor allowed the Associate Editor to decide the suitability for publication without sending it again to the reviewers, also in consideration of the given reviewer availability to re-evaluate the manuscript. In a few cases, a further revised manuscript was requested to the Authors in agreement to reviewers’ comments.

At the end of this process, 10 articles were accepted for publication as supplements in the BMC Bioinformatics journal. A short presentation of each of these contributions follows.

## BMC Bioinformatics supplement content

The articles accepted for publication in the BMC Bioinformatics supplement devoted to BITS 2015 cover different aspects of the field, from novel algorithms, applications and comparisons of analysis methods to specific data, and tool developments.

Nuzzo A. et al. [2]. Title: KAOS: a new automated computational method for the identification of overexpressed genes.

The authors suggest a user-friendly tool called KAOS (Kinase Automatic Outliers Search). This is a new automated computational method for the identification of overexpressed genes. In this paper the authors propose a new computational method for the identification of genes which are selectively over-expressed in a very small fraction of samples within a specific tissue. The authors focus on a test set of Kinase over-expression and activation as a consequence of gene amplification or gene fusion events, which are well-known mechanisms of tumorigenesis.

Zucca S. et al. [3]. Title: Analysis of amplicon-based NGS data from neurological disease gene panels: a new method for allele drop-out management.

The authors present a new procedure to manage sequencing artifacts attributable to PCR-like amplification in the next-generation sequencing applications. This methodology has been compared to the Illumina Custom Amplicon workflow, available on Illumina MiSeq, regarding the analysis of data obtained with four newly designed TruSeq Custom Amplicon gene panels. In particular this method shows to manage one of these complex configurations, when two point mutations occur, and it is platform-independent.

Valle I. F. et al. [4]. Title: Optimized pipeline of MuTect and GATK tools to improve the detection of somatic single nucleotide polymorphisms in whole-exome sequencing data.

In this paper the authors describe a new method based on a pipeline able to detect a wide profile of single nucleotide mutations with high validation rates. This pipeline is able to combine the output of two standard tools – Genome Analysis Toolkit (GATK) and MuTect – and adapt their algorithms to create the GATK-LODN method. The GATK-LOD_N_ increases the performance of the GATK variant detection, while preserving mutations not detected by MuTect. Experiments in simulated data demonstrated that GATK-LODN increased both specificity and sensitivity of GATK results.

Pellegrini M. et al. [5]. Title: Protein complex prediction for large protein-protein interaction networks with the Core&Peel Method.

In this work, the authors apply a specific clustering algorithm for predicting protein complexes (PC) in large protein-protein interaction networks (PPIN). This method provides an algorithmic solution with polynomial running time which guarantees good output quality on challenging large protein-protein interaction networks. In the test set used, the authors demonstrate that it outperforms ten competitors in its ability to identify known protein complexes and in the functional coherence of its predictions.

Manconi A. et al. [6]. Title: Removing duplicate reads using Graphics Processing Units.

In this work the authors present the new method “GPU-DupRemoval”, a GPU based method to remove identical and nearly identical duplicates generated with Illumina platform. This method implements a prefix-suffix comparison approach and is able to cluster the reads without constraints on the maximum length of the prefixes, supports both single- and paired-end read libraries, and is capable to analyse large clusters of potential duplicates. The authors show that this method outperforms most of cutting-edge solutions in terms of speed and amount of duplicates reads.

Diroma M. A. et al. [7]. Title: A comprehensive collection of annotations to interpret sequence variation in human mitochondrial transfer RNAs.

In this paper the authors present the “MToolBox“ tool, which is to be used for mitochondrial DNA analysis of high throughput next generation sequencing data. It allows to identify and characterize relevant variants not only in protein coding regions, but also in tRNA genes, by integrating tRNA variant annotations. An accurate annotation of mitochondrial tRNA variants can be used by mitochondrial researchers and clinicians to discover the functional role of tRNA variations. The authors provide both the stand-alone version and the web-based tool at the Mitochondrial Disease Sequence Data Resource (MSeqDR) website.

Cangelosi D. et al. [8]. Title: Artificial Neural Network classifier predicts neuroblastoma patients’ outcome.

In this paper the authors present a robust classifier based on an Artificial Neural Network classifier to predict the neuroblastoma patients’ outcome. The classifier uses the hypoxia gene expression signature (NB-hypo). The prediction was accurate in assessing the death of five low/intermediated risk patients and, by using the GSEA analysis of tumor gene expression profile, the authors where also able to demonstrate the hypoxic status of the tumor in patients with worse prognosis.

Consiglio A. et al. [9]. Title: A fuzzy method for RNA-Seq differential expression analysis in presence of multireads.

In this work the authors present an innovative approach based on fuzzy set theory to deal with multireads and evaluate differential expression events in high-throughput RNA sequencing data. This method is able to manage the uncertainty in gene expression estimation by defining the fuzzy read counts and to evaluate the possibility of a gene to be differentially expressed by using the over-expression, same-expression and under-expression fuzzy concepts. This method can be used to compute reliable differential expression events and to highlight possible false positives.

Cava C. et al. [10]. Title: How interacting pathways are regulated by miRNAs in breast cancer subtypes.

In this paper the authors introduce a new method to reveal miRNAs able to regulate, in a coordinated way, networks of gene pathways involved in subtypes of breast cancer. The method uses the pairs of functionally related pathways to create a network of dependent pathways used to identify miRNAs that could act as miRNA drivers in a coordinated process of regulation.

Granata I. et al. [11]. Title: Var2GO: a web-based tool for gene variants selection.

In this work the authors introduce “Var2GO”, a new web-based tool to support the annotation and filtering of genes coming from variant calling of next generation high-throughput sequencing data. The web server Var2GO permits to upload the variants’ table into a temporary database. All the genes associated with the variants are annotated with the corresponding Gene Ontology terms covering all the GO domains: Molecular function, Cellular component and Biological process. Based on this web tool approach, biologists can focus their search only on relevant genes coming from variant calling analysis, filtering for the relevant terms in their search.
